# A Robotic Pancreaticoduodenectomy Case Involving a Meandering Main Pancreatic Duct Coursing Posterior to a Replaced Common Hepatic Artery and in Front of the Portal Vein

**DOI:** 10.70352/scrj.cr.24-0184

**Published:** 2025-03-21

**Authors:** Yui Sawa, Yosuke Inoue, Kosuke Kobayashi, Atsushi Oba, Yoshihiro Ono, Hiromichi Ito, Yu Takahashi

**Affiliations:** Division of Hepato-Biliary-Pancreatic Surgery, Cancer Institute Hospital, Japanese Foundation for Cancer Research, Tokyo, Japan

**Keywords:** pancreas, pancreaticoduodenectomy, meandering main pancreatic duct, replaced common hepatic artery, annular pancreas

## Abstract

**INTRODUCTION:**

Anatomical anomalies, such as branches of the celiac artery and superior mesenteric artery (SMA), and pancreatic malformations, including the annular pancreas, are important during pancreaticoduodenectomy (PD). Here, we report a case of an anomaly of the artery and main pancreatic duct (MPD) in which the pancreatic parenchyma surrounded the replaced common hepatic artery (rCHA), and the meandering main pancreatic duct (MMPD) ran behind the rCHA.

**CASE PRESENTATION:**

A 71-year-old woman was diagnosed with intraductal papillary mucinous neoplasm (IPMN) of pancreatic body and the dilation of MPD to 13 mm, which was a factor of high-risk stigmata. Preoperative computed tomography (CT) showed that the rCHA branched from the superior mesenteric artery (SMA) and the pancreatic parenchyma surrounded the rCHA. Moreover, the MPD meandered and ran behind the rCHA. PD was performed. At the time of dissection between the rCHA and pancreatic parenchyma, we had to divide not only the cranial part of the pancreatic parenchyma along the rCHA but also the MPD. The postoperative course was uneventful.

**CONCLUSION:**

This is the first report of the rCHA surrounded by pancreatic parenchyma and MMPD running behind the rCHA and in front of the portal vein.

## Abbreviations

CT
: computed tomography
d-rCHA
: distal replaced common hepatic artery
IPMN
: intraductal papillary mucinous neoplasm
MMPD
: meandering main pancreatic duct
MPD
: main pancreatic duct
MRI
: magnetic resonance imaging
PD
: pancreaticoduodenectomy
p-rCHA
: proximal replaced common hepatic artery
PV
: portal vein
rCHA
: replaced common hepatic artery
SMA
: superior mesenteric artery
SMV
: superior mesenteric vein
SpV
: splenic vein

## INTRODUCTION

Pancreatoduodenectomy (PD) has various sections of resection and dissection around major vessels, making the procedure complicated.^1)^ Therefore, understanding the anatomy using computed tomography (CT) and magnetic resonance imaging (MRI) before surgery is important to improve the quality of PD procedures. Anatomical anomalies, such as the branches of the celiac artery and superior mesenteric artery (SMA), and pancreatic malformations, including the annular pancreas, are important during PD.^2–5)^ All of these anomalies have been reported in some past reports^6–8)^ and can significantly affect surgical procedures.^9,10)^ Here, we report a novel case of an anomaly of the artery and main pancreatic duct (MPD), in which the pancreatic parenchyma surrounded the replaced common hepatic artery (rCHA) and the meandering main pancreatic duct (MMPD) ran behind the rCHA and in front of the portal vein.

## CASE PRESENTATION

**Fig. 1:**
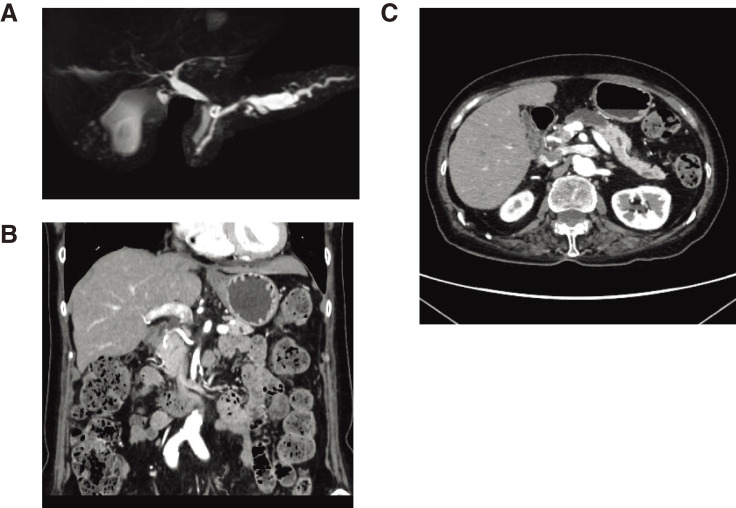
Preoperative imaging findings. (**A**) Magnetic resonance cholangiopancreatography shows the dilation of the main pancreatic duct (MPD) and the meandering MPD. (**B**) Computed tomography (CT) shows the replaced common hepatic artery (rCHA) surrounded by the pancreas. (**C**) CT shows that the MPD runs behind the rCHA.

A 71-year-old woman was diagnosed with a branched intraductal papillary mucinous neoplasm (IPMN) of the pancreatic body. She was regularly followed up at a hospital for IPMN. MRI revealed no signs of high-risk stigmata until 2023. However, in 2024, MRI revealed a rapid dilation of the MPD. Endoscopic ultrasonography also showed dilation of the MPD and a contrast-enhanced mural nodule 10 mm in size in the proximal area of the IPMN. A biopsy performed during the endoscopic retrograde cholangiopancreatography revealed cellular atypia. The patient was referred to our hospital for treatment. In our hospital, CT and MRI revealed IPMN of pancreatic body and the dilation of MPD to 13 mm, which was a factor of high-risk stigmata in IPMN diagnosis (**Fig. 1A**; **Supplementary Video 1**). Her height and weight were 161.8 cm and 57.65 kg, respectively. The carcinoembryonic antigen: 1.9 ng/mL, carbohydrate antigen 19-9: 4.1 U/mL, duke pancreatic monoclonal antigen type 2: 25 U/mL, s-pancreas-1 antigen: 6.8 U/mL, hemoglobin: 13.7 g/dL, leukocytes: 6730/µL, c-reactive protein: 0.04 mg/dL, creatinine: 0.68 mg/dL, asparate aminotransferase: 20 U/L, alanine aminotransferase: 19 U/L, total bilirubins: 0.7 mg/dL, amylase: 38 U/L, platelets: 201000/µl, prothrombin time international normalized ratio: 0.80, and hemoglobin A1c: 6.9%. She had a history of pulmonary tuberculosis, Sjögren syndrome, colonic polyps, and facial paralysis. The American Society of Anesthesiologists physical status classification system score was 2. Preoperative CT showed that the rCHA branched from the SMA and that the pancreatic parenchyma surrounded the rCHA (**Fig. 1B**, **Supplementary Videos 2, 3**). The main pancreatic duct meandered and ran behind the rCHA (**Fig. 1C**, **Supplementary Videos 2, 3**).

**Fig. 2:**
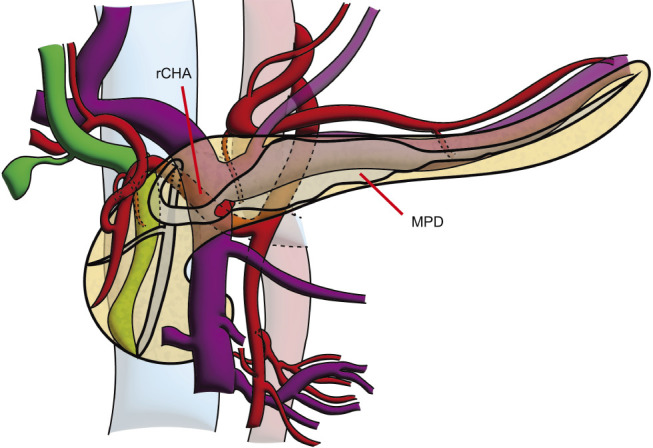
Intraoperative schema. The replaced common hepatic artery (rCHA) is surrounded by the pancreas, and the MPD runs behind the rCHA and in front of the portal vein.

**Fig. 3:**
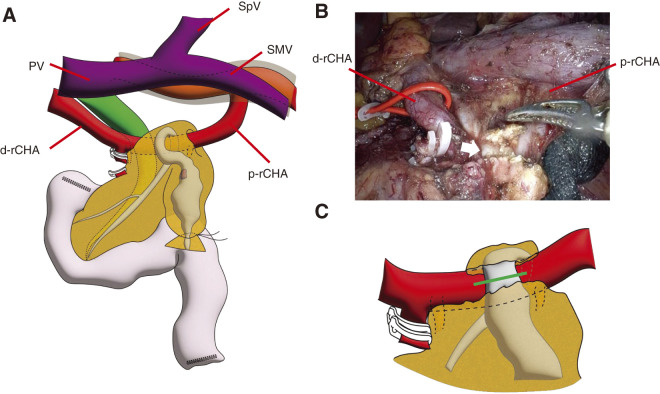
Intraoperative schema and picture. (**A**) The main pancreatic duct (MPD) runs over the replaced common hepatic artery (rCHA) with thin pancreas parenchyma. (**B**) The MPD (white arrow) runs over the taped rCHA. (**C**) The MPD was cut with the green line and the rCHA was dissected from the pancreas. PV, portal vein; SpV, splenic vein; SMV, superior mesenteric vein; d-rCHA, distal rCHA; p-rCHA, proximal rCHA

To maximize the surgical margin of the nodular lesion in the IPMN, robotic pancreaticoduodenectomy with D1 lymph node dissection was planned and performed. The operative time was 442 min and the estimated blood loss was 50 mL. The left posterior approach was used for SMA dissection, and the root of the rCHA was identified on the left side.^11–13)^ Pancreas transection was performed with the transection line positioned to the left of the SMA at the point the dilation of MPD normalized. Intraoperative frozen sections confirmed that the pancreatic stump was negative for carcinoma. During dissection of the upper side of the duodenum, the peripheral part of the rCHA was identified, and the gastroduodenal artery branching from the rCHA was dissected. At the time of dissection between the rCHA and pancreatic parenchyma, we divided the cranial part of the pancreatic parenchyma along the rCHA and cut the MPD again (**Figs. 2** and **3**). The main pancreatic duct was clipped bilaterally and divided to excavate the rCHA. Two thin arterial branches extending from the rCHA to the pancreas were identified, ligated, and divided. The remaining steps were performed as usual (**Supplementary Video 4**).

Postoperative course was good. There was a postoperative pancreatic fistula of Grade1 (Clavien–Dindo classification) and biological leakage (International Study Group of Pancreatic Surgery classification). The patient was discharged on the 13th day after the operation. The pathological findings included intraductal papillary mucinous carcinoma, noninvasive nature of the pancreatic body, and no lymph node metastases.

## DISCUSSION

Anomalies of the celiac artery and SMA branches have been reported in previous articles.^14,15)^ In these cases, the case had rCHA, which are seen in 1.5%–2.5% of all cases^7,8)^. Her rCHA was also intrapancreatic type and the intrapancreatic rCHA had only been reported in a small number of cases.^16,17)^ The rCHA was categorized as type A by Ha et al., wherein half of the rCHAs penetrated the pancreatic parenchyma.^18)^ The intrapancreatic hepatic artery is usually associated with the hepatomesenteric trunk, as was this case.^19)^

Our case had MMPD which was categorized as type B2, with an incidence of 2.2%.^20)^ Some reports and classifications of MPD anomalies in the portal annular pancreas are available.^21,22)^ There was one report of portal annular pancreas with the rCHA and one report of compression of the MPD by intrapancreatic rCHA running across the MPD supero-posteriorly.^23,24)^ However, there are no reports of the rCHA and MMPD being observed together, and there are no reports of the MMPD running behind the intrapancreatic rCHA and above the portal vein.

Our case had a minor complication of a pancreatic fistula (biochemical leakage). A previous study reported that the rCHA affects the postoperative outcome of PD.^25)^ There were also reports that MMPD can cause acute pancreatitis,^20,26,27)^ and MMPD can be found more often in case of IPMN than in others.^28)^ However, there have been no reports of MMPD related to surgery.

It has been reported that MMPD originates from a sequence of pancreatic fusions during its developmental stage.^29)^ Usually, the ventral pancreas joins the dorsal pancreas at the pancreatic head, and the Wirsung duct of the ventral pancreas joins the Santorini duct of the dorsal pancreas to form the main pancreatic duct.^30)^ In this case, the Santorini duct initially courses between the rCHA and the portal vein and fuses dorsally with the Wirsung duct.

In this anomaly, when the pancreas and the rCHA are divided, the pancreatic parenchyma, including the MPD, must be dissected at the head of the rCHA. The same technique was used for this procedure. There was no dilation of the MPD at this location and clipping of the MMPD was performed, which was considered oncologically permissible. We encountered a rare case in which an MMPD ran behind the rCHA and surrounded the pancreatic parenchyma. With a close review of preoperative images, this anatomical anomaly can be recognized and surgery can be safely performed.

## CONCLUSION

This is the first report of the rCHA surrounded by pancreatic parenchyma and MMPD running behind the rCHA and in front of the portal vein.

## DECLARATIONS

### Funding

The authors received no specific funding for this work.

### Author’s contribution

YS joined the surgical procedures, data interpretation, and preparation of the manuscript.

YI performed the surgical procedures, data interpretation, and preparation of the manuscript.

KK, AO, YO, HI, and YT contributed to the discussion. All authors have read and approved the manuscript.

### Availability of data and materials

Data will be made available on reasonable request.

### Ethics approval and consent to participate

This report has been performed in accordance with the Declaration of Helsinki, and was approved by the ethical review board of Cancer Institute Hospital, Japanese Foundation for Cancer Research (2023-GB-100). Consent for participation in this study of this case report was also obtained from the patient.

### Consent for publication

Informed consent to publish has been obtained from the patient.

### Competing interests

The authors of this manuscript have no conflicts of interest to disclose described by the *Surgical case reports*.

## SUPPLEMENTARY MATERIALS

Supplementary Video 1

Supplementary Video 2

Supplementary Video 3

Supplementary Video 4
